# Supplementary figures and images for: Immune Landscape of Invasive Ductal Carcinoma Tumor Microenvironment Identifies a Prognostic and Immunotherapeutically Relevant Gene Signature
Source: Front Oncol. 2019 Sep 18;9:903. doi: 10.3389/fonc.2019.00903 (PMC6759595; doi:10.3389/fonc.2019.00903)

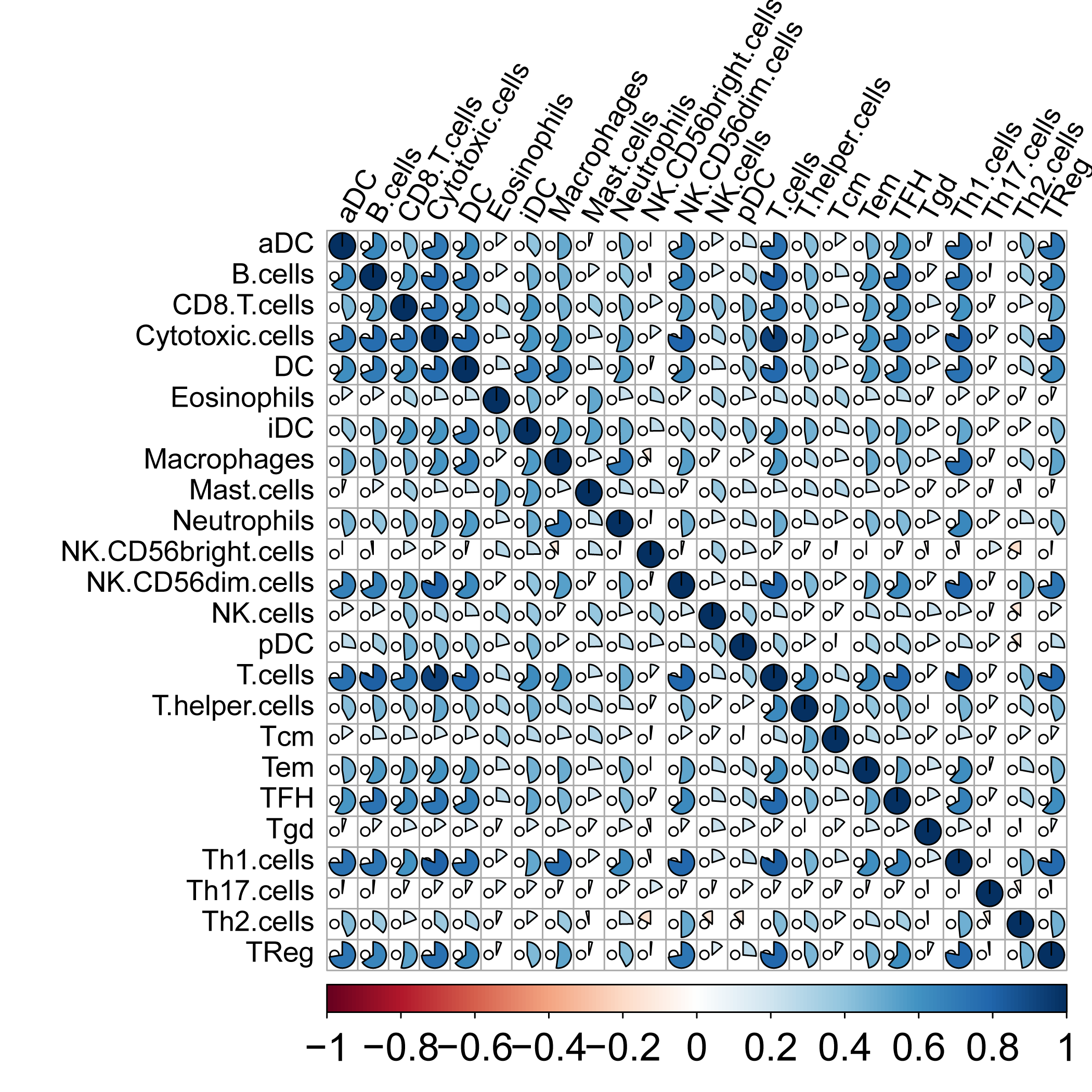

Supplement: Figure S1 — The correlation between different infiltrating immune cells. [file Image_1.TIF]

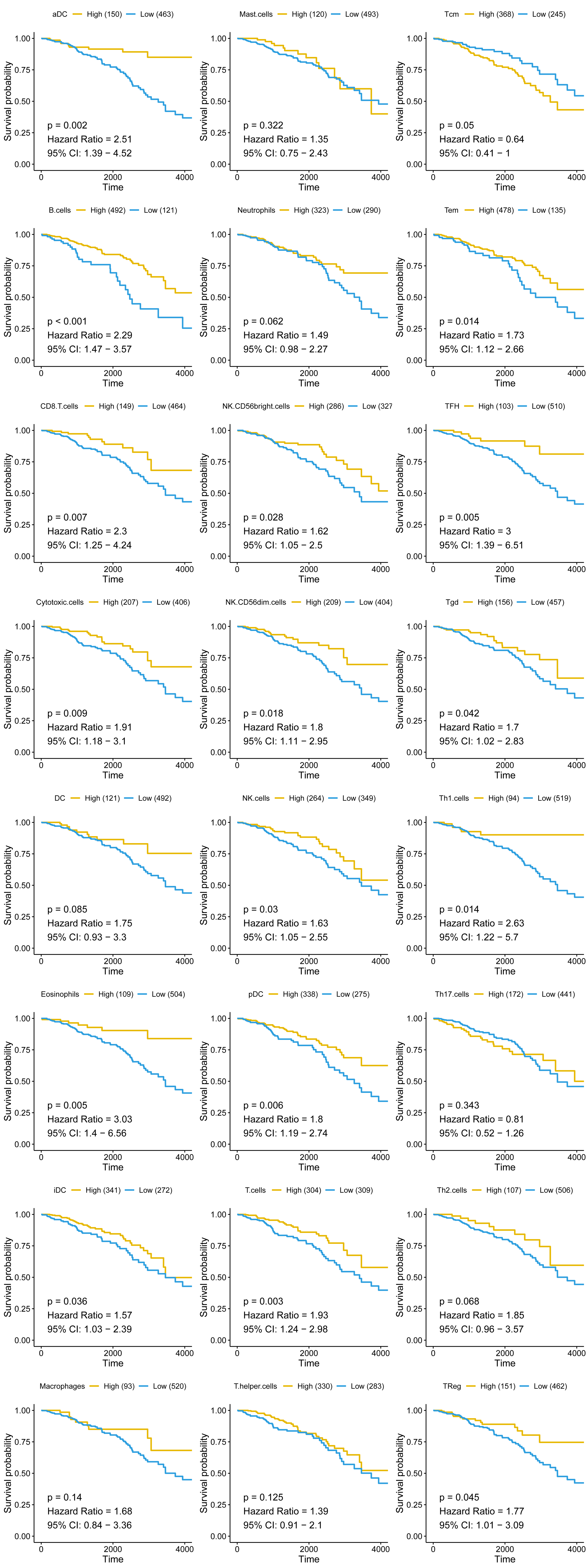

Supplement: Figure S2 — The correlation between the ssGSEA scores of infiltrating immune cells and the OS probability of IDC patients. [file Image_2.TIF]

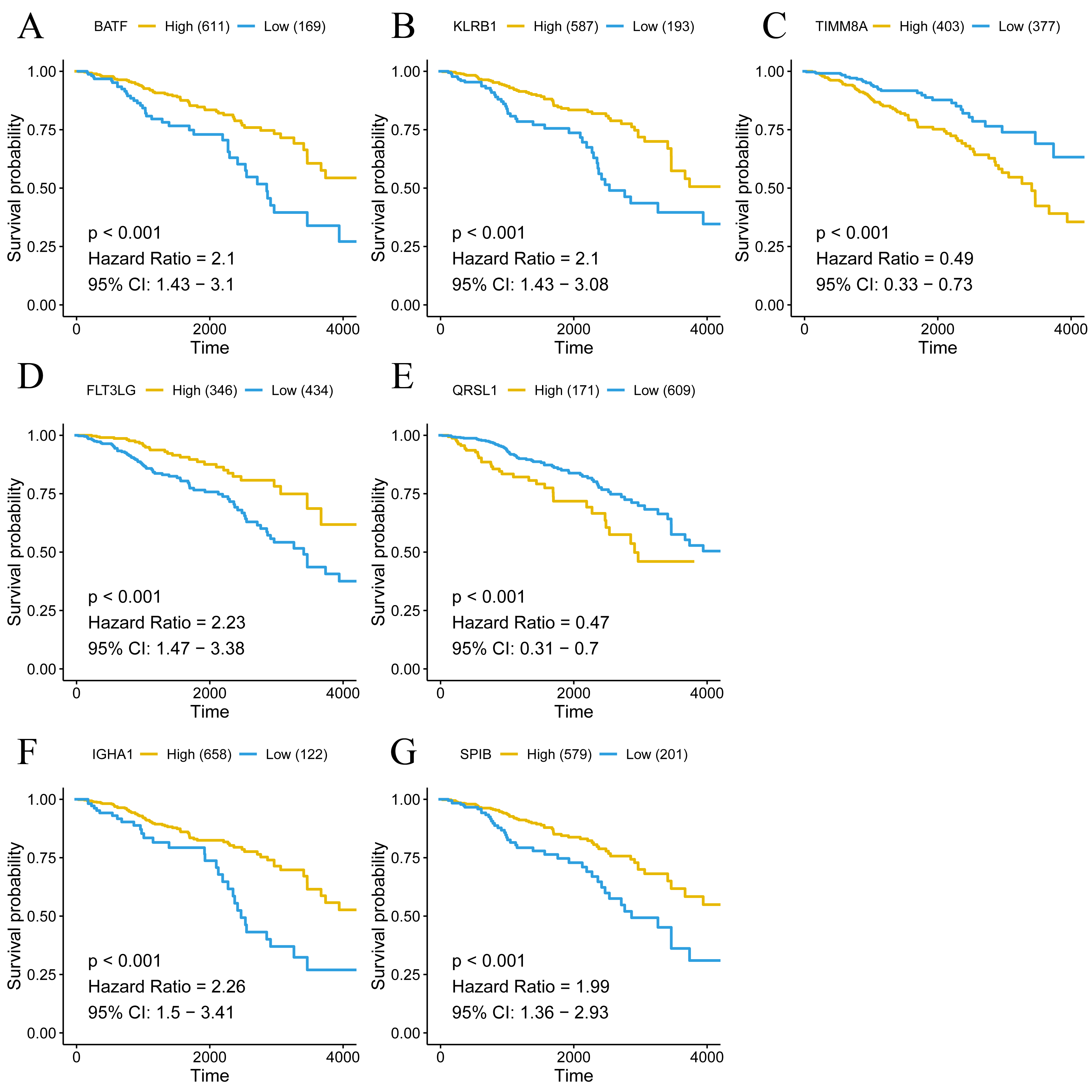

Supplement: Figure S3 — The correlation between the expression level of seven genes in the immune signature and the OS probability of IDC patients. (A) BATF, (B) KLRB1, (C) TIMM8A, (D) FLT3LG, (E) QRSL1, (F) IGHA1, (G) SPIB. [file Image_3.TIF]

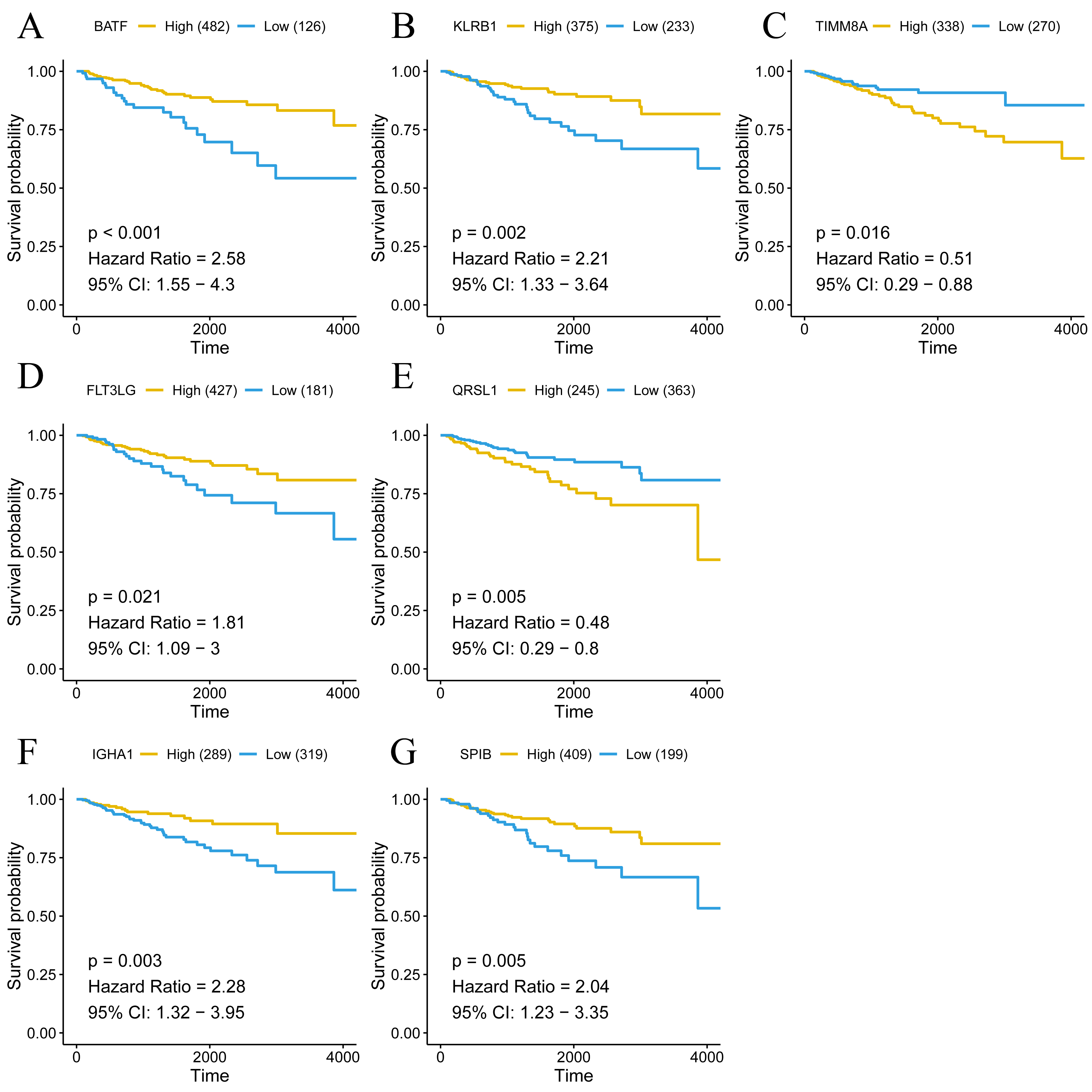

Supplement: Figure S4 — The correlation between the expression of seven genes in the immune signature and the RFS probability of IDC patients. (A) BATF, (B) KLRB1, (C) TIMM8A, (D) FLT3LG, (E) QRSL1, (F) IGHA1, (G) SPIB. [file Image_4.TIF]

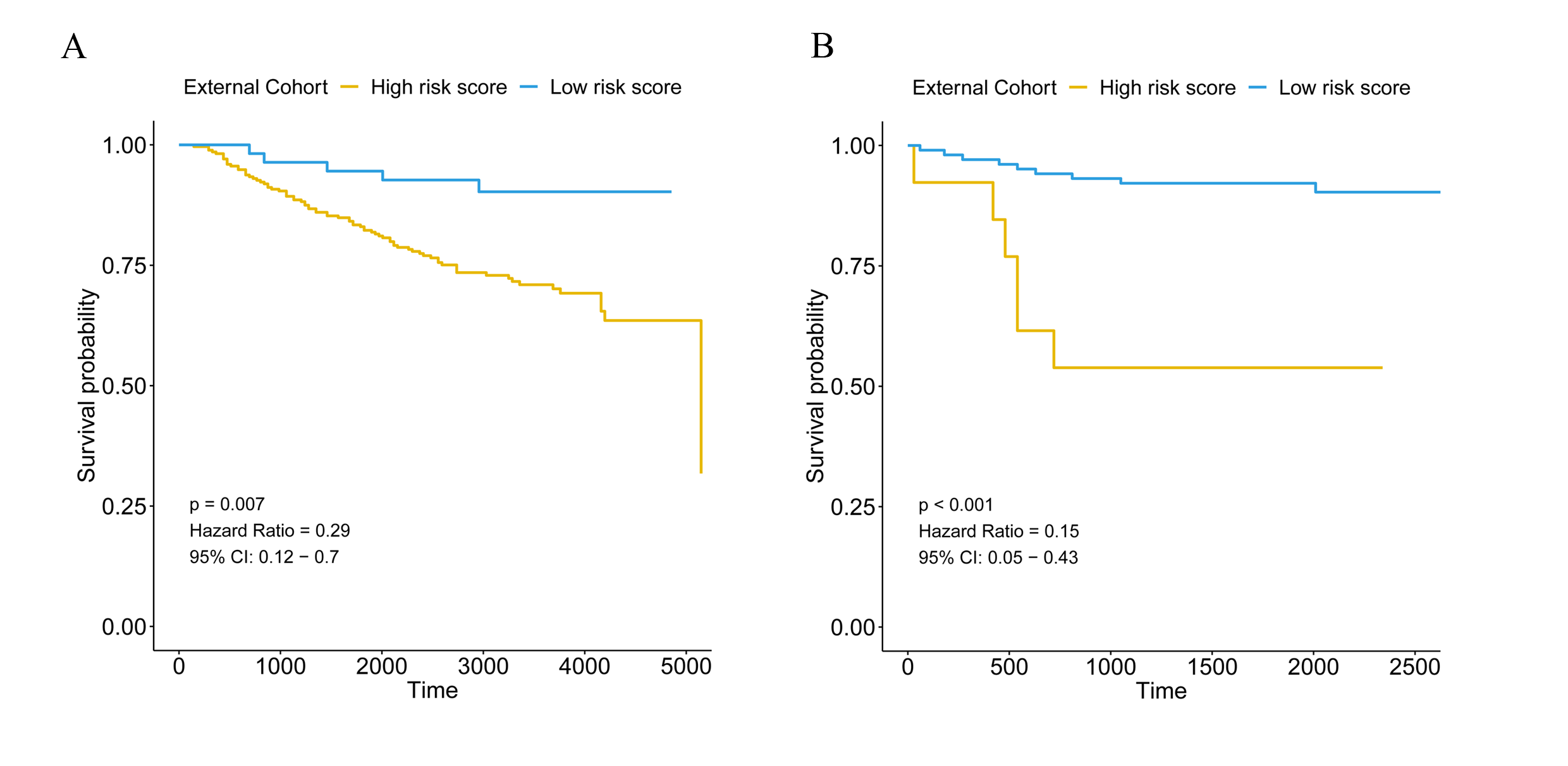

Supplement: Figure S5 — Validation of the immune signature in two external cohorts, GSE20685 (A) and GSE86948 (B). [file Image_5.TIF]

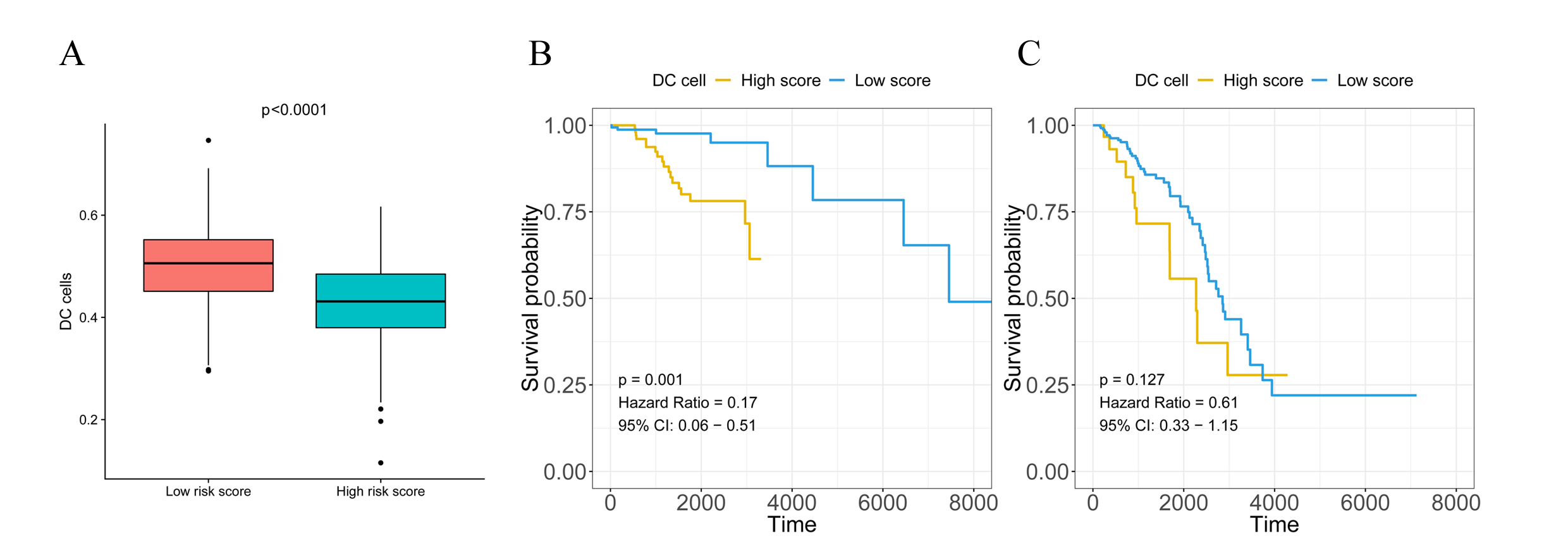

Supplement: Figure S6 — The correlation between the ssGSEA scores of DCs and the OS probability of IDC patients in the high- and low-risk score groups. (A) The ssGSEA scores were higher in the high- and low-risk score groups. (B) The correlation between the ssGSEA scores of DCs and the OS probability of IDC patients in the low-risk score group. (C) The correlation between the ssGSEA scores of DCs and the OS probability of IDC patients in the high-risk score group. [file Image_6.TIF]

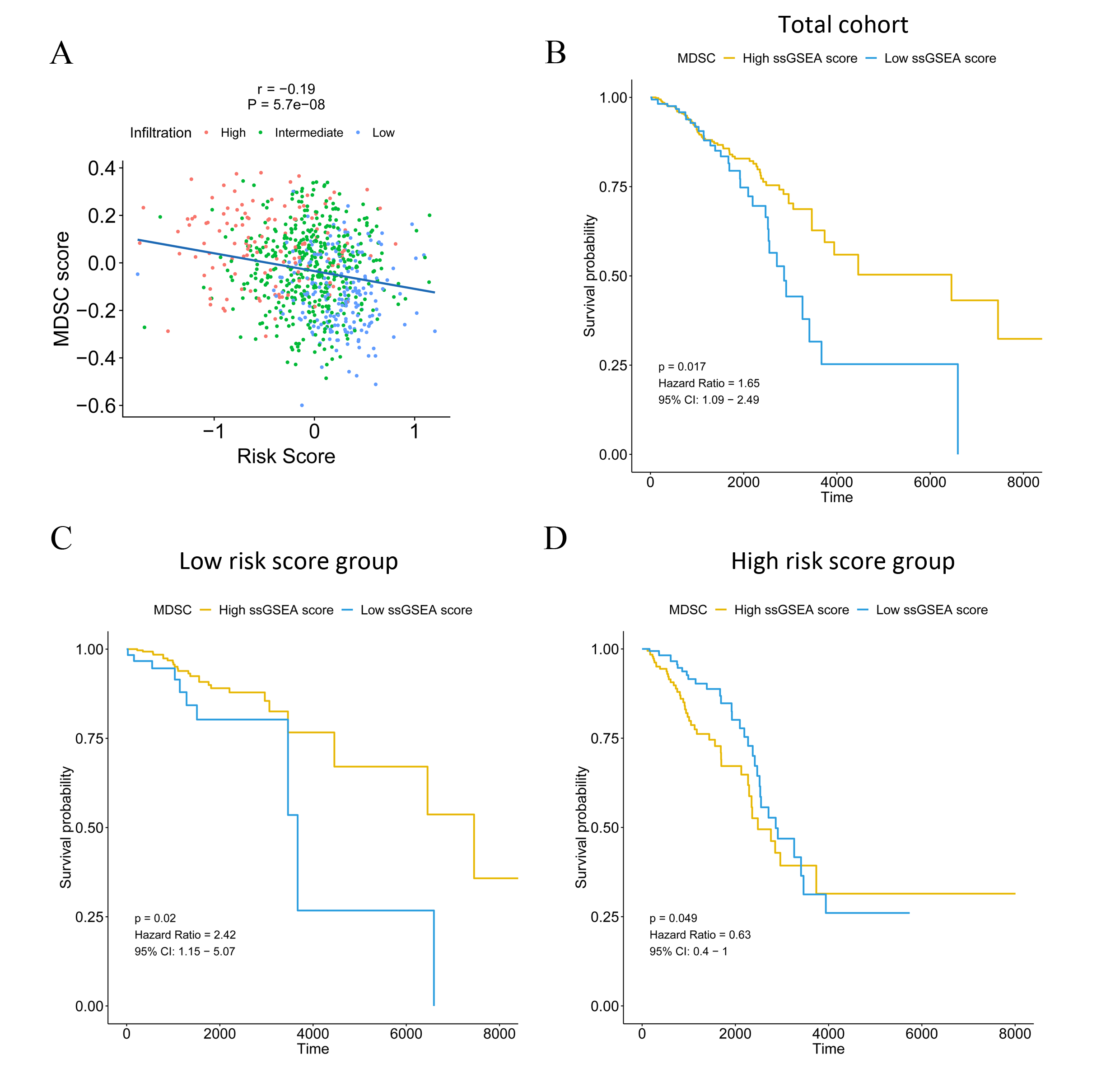

Supplement: Figure S7 — The correlation between the ssGSEA scores of MDSCs and the OS probability of IDC patients in the high- and low-risk score groups. (A) The correlation between MDSC ssGSEA scores and risk scores. (B) The correlation between the ssGSEA scores of DCs and the OS probability of IDC patients in the whole cohort. (C) The correlation between the ssGSEA scores of DCs and the OS probability of IDC patients in the low-risk score group. (D) The correlation between the ssGSEA scores of DCs and the OS probability of IDC patients in the high-risk score group. [file Image_7.TIF]

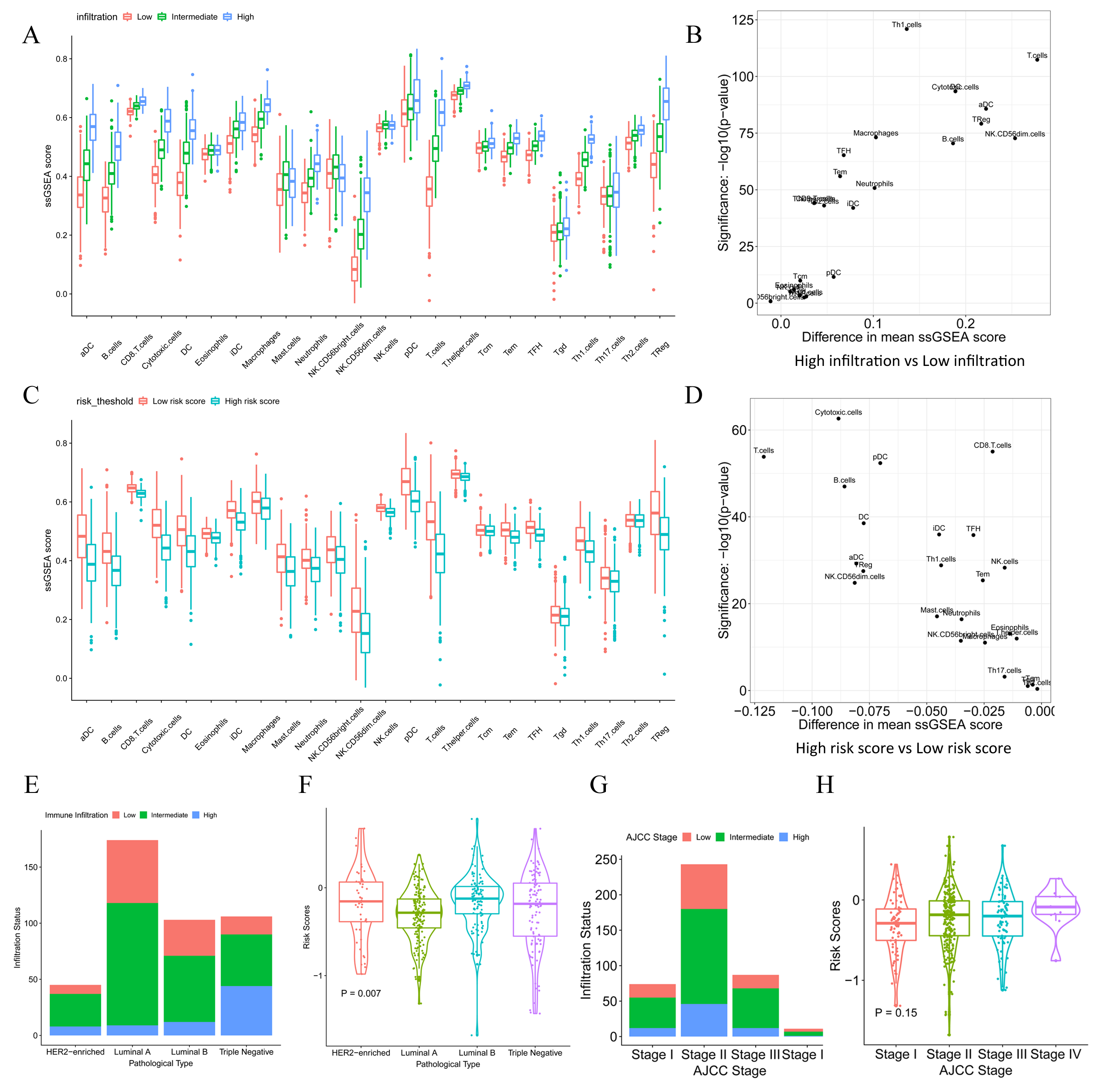

Supplement: Figure S8 — The ssGSEA score distribution in the low, intermediate, and high immune infiltration patterns and in the low- and high-risk score groups. (A) The ssGSEA score distribution in low, intermediate and high immune infiltration patterns. (B) The difference and P-value from the comparison between the ssGSEA score from low and high immune infiltration patterns. (C) The ssGSEA score distribution in the low- and high-risk score groups. (D) The difference and P-value from the comparison between the ssGSEA score from the low- and high-risk score group. (E) The distribution of immune infiltration patterns in different pathological subtypes. (F) The distribution of risk scores in different pathological subtypes. (G) The distribution of immune infiltration patterns at different pathological stages. (H) The distribution of risk scores at different pathological stages. [file Image_8.TIF]

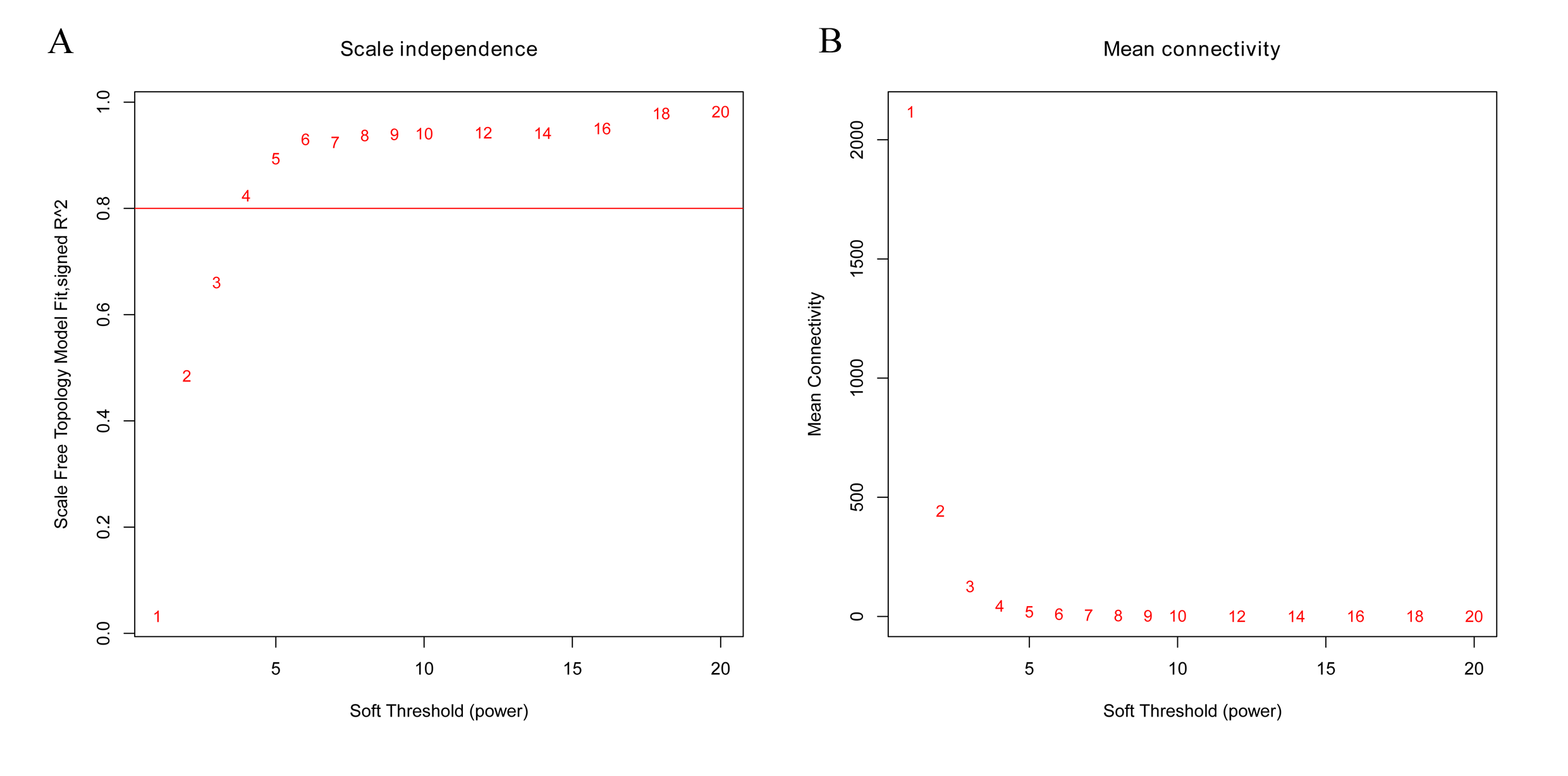

Supplement: Figure S9 — The selection of the soft threshold in the WGCNA. (A) Scale free topology model fit, (B) mean connectivity. [file Image_9.TIF]

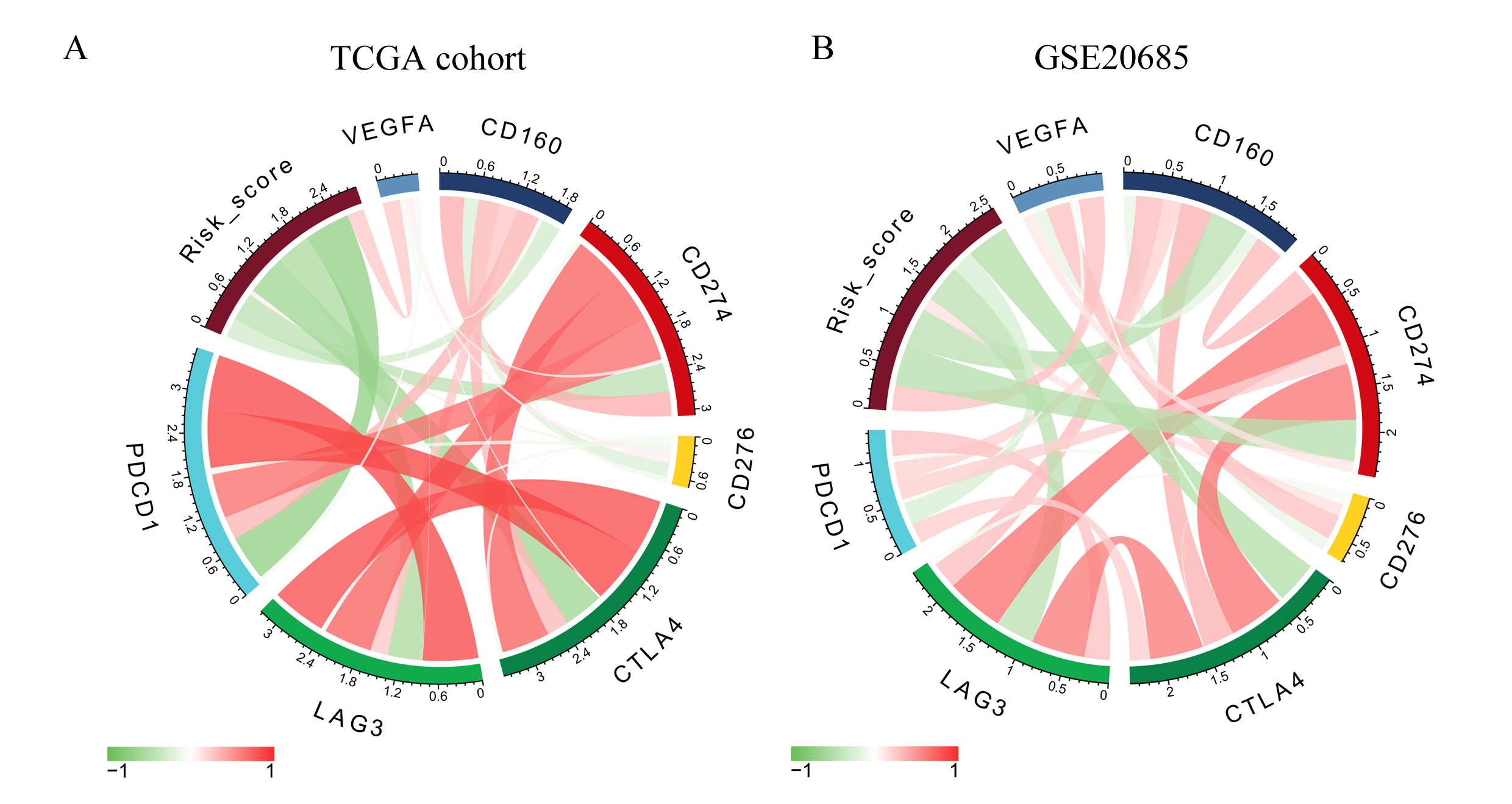

Supplement: Figure S10 — The correlation of the expression profiles of several immune checkpoint proteins, risk score, and VEGF-A in the TCGA (A) cohort and GSE20685 cohort (B). [file Image_10.TIF]
